# Neutrophil Extracellular Traps Facilitate A549 Cell Invasion and Migration in a Macrophage-Maintained Inflammatory Microenvironment

**DOI:** 10.1155/2022/8316525

**Published:** 2022-01-06

**Authors:** Lemeng Zhang, Huifang Yi, Jianhua Chen, Haitao Li, Yongzhong Luo, Tianli Cheng, Hua Yang, Zhou Jiang, Changqie Pan

**Affiliations:** Thoracic Medicine Department 1, Hunan Cancer Hospital, Changsha, Hunan Province 410013, China

## Abstract

**Introduction:**

The biological functions of neutrophil extracellular traps (NETs) in tumorigenesis have drawn an increasing amount of attention. This study explored the relationship between NETs and the inflammatory microenvironment in lung cancer cell invasion and metastasis.

**Methods:**

NETs were quantified using myeloperoxidase (MPO–DNA) and immunofluorescence staining. Cytokine levels were measured using ELISA kits. THP-1 and A549 cells were used for in vitro experiments. Transwell and Matrigel assays were used to assess the invasion and migration abilities of the cells.

**Results:**

Neutrophil infiltration and NET formation were observed in the lung cancer tissues. Compared with healthy controls, the level of MPO–DNA complexes in lung cancer patients increased remarkably and was positively correlated with peripheral blood neutrophil counts, smoking status, and poor prognosis. Increased circulating NET levels were also positively correlated with the levels of inflammatory cytokines, including IL-1*β*, IL-6, IL-18, and TNF-*α*. Neutrophils isolated from patients with lung cancer are more prone to NET release. NETs can promote the invasion and migration ability of THP-1 and A549 cell in coculture systems, while pretreatment with NET inhibitors can effectively reduce NET-induced invasion and metastasis. The ability of NETs to promote invasion and metastasis is partly dependent on macrophages.

**Conclusion:**

Taken together, our study demonstrated that NETs facilitate A549 cell invasion and migration in a macrophage-maintained inflammatory microenvironment.

## 1. Introduction

Lung cancer is the most commonly diagnosed malignant tumor and the main cause of cancer-related deaths worldwide, with estimates of 2.2 million new confirmed cases and 1.79 million deaths every year [1]. Despite the dramatic progress in targeted therapy and immunotherapy, the morbidity and mortality of lung cancer remain the highest among all malignant diseases, and the 5-year survival rate of patients with all stages of stage lung cancer is about 21%, compared with 6% for patients with distant metastases [2]. Emerging studies have shown that the main causes of lung cancer, such as smoking, chronic obstructive pulmonary diseases, and air pollution, are accompanied by a persistent chronic inflammatory microenvironment, in which inflammatory cells and cytokines affect the development and progression of cancer by promoting immune escape, tumor angiogenesis, epithelial mesenchymal transformation, and apoptosis [3, 4]. The study has illustrated that tobacco smoke and other irritants act as tumor promoters by initiating chronic inflammation [5]. Exposure to respirable particulate matter such as asbestos may activate pyrin domain-containing protein 3 (NLRP3) inflammasome and increase the risk of malignant mesothelioma and lung cancer [6]. In addition, inflammation and the inflammatory microenvironment can also function in the tumor response to chemotherapy drugs and hormones, thereby affecting the prognosis of tumors to a certain extent [7]. Therefore, further study of inflammatory mechanisms associated with lung cancer is of vital importance for anti-inflammatory prevention and cancer treatment.

Aside from the first line of antimicrobial defense, the functions of neutrophils in systemic inflammation have received substantial attention [8]. In response to infection or inflammatory response, activated neutrophils positively release extracellular chromatin and form an extracellular network structure called neutrophil extracellular traps (NETs). The composition of NETs is nonspecific, and NETs have proinflammatory properties that cause damage to surrounding tissues by increasing the proinflammatory response [9]. NETs can trap circulating cancer cells and promote tumor metastasis [10]. A mouse model-based study suggested that the formation of NETs induced by persistent inflammation is essential to awaken dormant cancer [11]. Additionally, NET formation can be induced by cathepsin C by mediating IL-1*β* to support the metastatic growth of cancer cells in the lungs [12]. Lee et al. constructed a mathematical model and found that NET-mediated invasion of lung cancer cells is related to neutrophil density and NET concentration [13]. According to another study, lung cancer cells can activate NETs, and extracellular RNAs are involved in this process [14]. Furthermore, our previous studies have found that neutrophils release NETs, leading to macrophage activation and systemic inflammatory responses during infection and hyperventilation [15, 16]. However, whether NETs contribute to the interaction between tumor-associated macrophages and neutrophils is still largely unknown.

In the present study, we explored the relationship between circulating NETs, clinical characteristics, and prognosis of patients with lung cancer. Additionally, we hypothesized that the release of NETs may contribute to inflammatory responses through the activation of macrophages, thus promoting lung cancer cell invasion and migration. Exploring the relationship and mechanisms between NETs, the inflammatory microenvironment, and lung cancer cell invasion and metastasis may provide new targets for the prevention and treatment of lung cancer.

## 2. Materials and Methods

### 2.1. NET Quantification

The myeloperoxidase–DNA (MPO–DNA) complexes were quantified using the Quant-iT PicoGreen double stranded DNA (dsDNA) assay kit (Invitrogen, USA) according to the manufacturer's instructions [17]. To quantify NETs in the cell culture supernatant and plasma, a capture ELISA of myeloperoxidase (MPO) associated with DNA was performed. For the capture antibody, an MPO kit (Hycult Biotech, HK210-01) was used.

### 2.2. Immunofluorescence Staining

The in situ structures of NETs in lung cancer tissues were detected as DAPI, neutrophil elastase (NE), and citrullinated histone-3 (citH3) colocalization. The lung cancer tissue and neutrophils were separately incubated with the specific primary antibodies for NE (1 : 100, BD Bioscience), citH3 (1 : 50; Abcam), and DAPI (1 : 800, Abcam). All slides were scanned under the same conditions for magnification, exposure time, lamp intensity, and camera gain. Confocal images were acquired using an Olympus Fluoview 1000 microscope (×40 with and without ×2.5 digital zoom).

### 2.3. Cytokine Quantification

We used commercial ELISA (R&D Systems, Minneapolis, MN, USA) to detect the levels of TNF-*α*, IL-1*β*, IL-6, and IL-18 in each sample. Briefly, the culture supernatant or the plasma was mixed with assay buffer as added to wells coated with anti-TNF-*α*, IL-1*β*, IL-6, or IL-18 antibodies at 37°C for 60 min. Then, HRP-conjugated monoclonal antibody was added and incubated at 37°C for 2 h, followed by incubation with colorimetric (tetramethylbenzidine) solution for another 10 min. We then measured the relative absorbance, and three independent experiments were performed to quantify the levels of each cytokine.

### 2.4. Neutrophil Isolation and NET Formation

Neutrophils were isolated immediately by a one-step gradient centrifugation method using Polymorph Prep (Axis-Shield, Dundee, United Kingdom). The neutrophil layer was isolated and resuspended in Roswell Park Memorial Institute (RPMI) 1640 medium supplemented with 10% fetal calf serum. Trypan blue exclusion showed that viability was 96% for all preparations. Neutrophils were cultured in 24-well tissue culture plates and incubated with inhibitors 30 min before stimulation, as per the manufacturer's instructions. Phorbol 12-myristate 13-acetate (PMA; 10 ng/ml) (Sigma-Aldrich, ON, USA) was added for NET formation. After 3 h of stimulation, cells were centrifuged and the cell-free supernatants were collected. The supernatants were stored at −20°C.

### 2.5. Coculture System

A549 (human lung adenocarcinoma cell line) and THP-1 cells (human peripheral blood monocytes) were obtained from American Type Culture Collection (Manassas, VA, USA). All cells were cultured in RPMI 1640 or Dulbecco's modified eagle medium (DMEM) supplemented with 10% FBS (Biowest, South America Origin), 100 U/ml penicillin sodium, and 100 mg/mL streptomycin sulfate at 37°C in a humidified atmosphere containing 5% CO2. This coculture system has been described previously [18].

### 2.6. Transwell and Matrigel Assays

Transwell and Matrigel assays were performed. Briefly, A549 and THP-1 cell lines were seeded into Matrigel-coated (for the invasion assay) or uncoated (for the migration assay) upper chambers of Transwell (BD Biosciences). The cells were incubated overnight, and noninvasive/migratory cells were removed. The cells that had transitioned to the membrane of the lower chamber were fixed for future analysis. An inverted microscope (Olympus Life Systems) was used to count the number of cells in five random fields per chamber.

### 2.7. Patients and Clinical Data Collection

This study included patients with lung cancer who underwent surgery at Hunan Cancer Hospital (Changsha, Hunan, China) between 2018 and 2020. All diagnoses were confirmed based on the pathological reports. The Ethics Committee of Hunan Cancer Hospital approved this study, and informed consent was obtained from all the study patients.

### 2.8. Statistical Analysis

The SPSS statistical package was used for all analyses (version 22.0; IBM Corp., Armonk, NY, USA). Data are presented as the mean ± standard deviation. The Kaplan-Meier method was used for the prognostic analysis. Spearman's correlation coefficient was used to calculate the correlation between circulating NET levels, circulating inflammatory cytokines, and peripheral blood neutrophil counts. A paired Student's *t*-test was used for two groups for normal distributions, while unpaired samples were compared using the Mann-Whitney *U* test, and statistical significance was set at *P* < 0.05.

## 3. Results

### 3.1. Neutrophil Infiltration and NET Formation Exist in Lung Cancer Tissues

**Figure 1:**
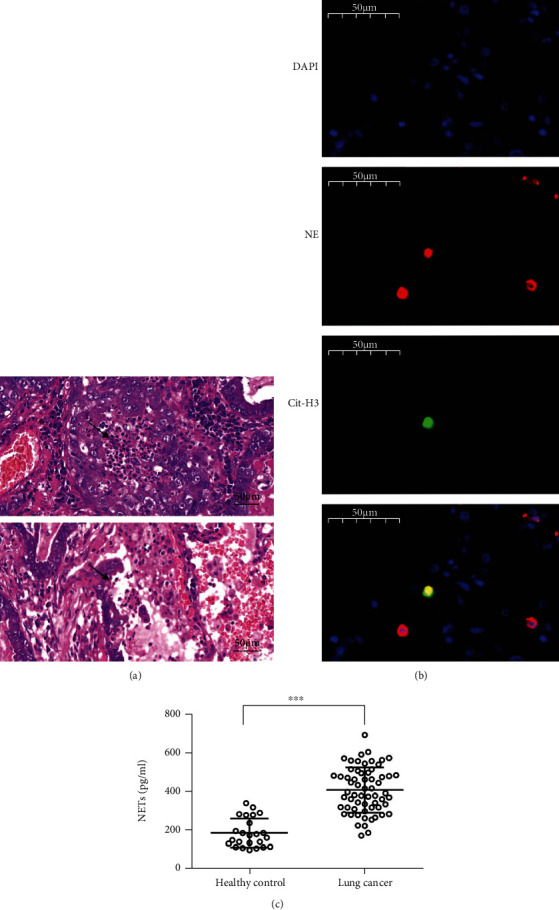
Neutrophil infiltration and NET formation exist in lung cancer tissues. (a) Neutrophil infiltration and (b) structure of NETs were demonstrated in postoperation pathological tissues from lung cancer patients. *In situ* structures of NETs in lung cancer tissues were detected as DAPI, neutrophil elastase (NE), and citrullinated histone-3 (citH3) colocalization. (c) The level of plasma MPO–DNA complexes in lung cancer patients and healthy controls. ^∗∗∗^*P* < 0.001.

Neutrophils have broad effects on the tumor microenvironment, including the release of oxidants and granular constituents, extracellular matrix remodeling, and cross signaling to inflammatory cells [19]. Neutrophil infiltration (Figure 1(a)) was demonstrated in postoperative pathological tissues from patients with lung cancer. NET structures (Figure 1(b)) were characterized by the colocalization of citH3 and NE with the NETs. To investigate the clinical application of NET release, we measured the concentration of circulating MPO–DNA complexes. Plasma samples were collected from twenty-four healthy individuals and sixty-two patients with lung cancer. The clinical characteristics, such as sex, age, TNM stage, pathological type, smoking status, peripheral blood neutrophil counts, and prognosis, were collected (Table 1). It is noteworthy that compared with the healthy controls, the levels of MPO–DNA complexes in lung cancer patients increased remarkably (Figure 1(c)).

### 3.2. High Levels of Circulating NETs Were Associated with Poor Prognosis

**Figure 2:**
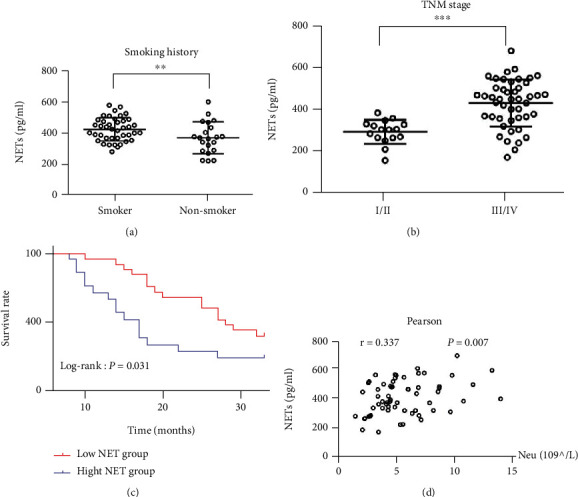
The correlation between circulating NET levels and clinical characteristics and prognosis of lung cancer patients. Increased circulating levels of NETs were positively correlated with (a) smoking status, (b) advanced clinical TNM stage, and (d) the peripheral blood neutrophil counts. The patients were divided into the high-NET and low-NET groups; (c) the high-NET group was associated with poor prognosis. ^∗∗^*P* < 0.01; ^∗∗∗^*P* < 0.001.

The correlation between circulating NET levels and the clinical characteristics of lung cancer patients was analyzed. Increased circulating levels of NETs were positively correlated with smoking status (Figure 2(a)), advanced clinical TNM stage (Figure 2(b)), and peripheral blood neutrophil counts (Figure 2(d)). The levels of circulating NETs were significantly increased in the heavy smokers, advanced lung cancer patients, and patients with increased peripheral blood neutrophil counts. Based on the median NET level as the cut-off value, the patients were divided into a group with high levels of NET and a group with low levels of NET. The group with high levels of circulating NET was associated with poor prognosis (Figure 2(c)).

### 3.3. The Level of Circulating NETs Was Associated with Inflammatory Cytokines

**Figure 3:**
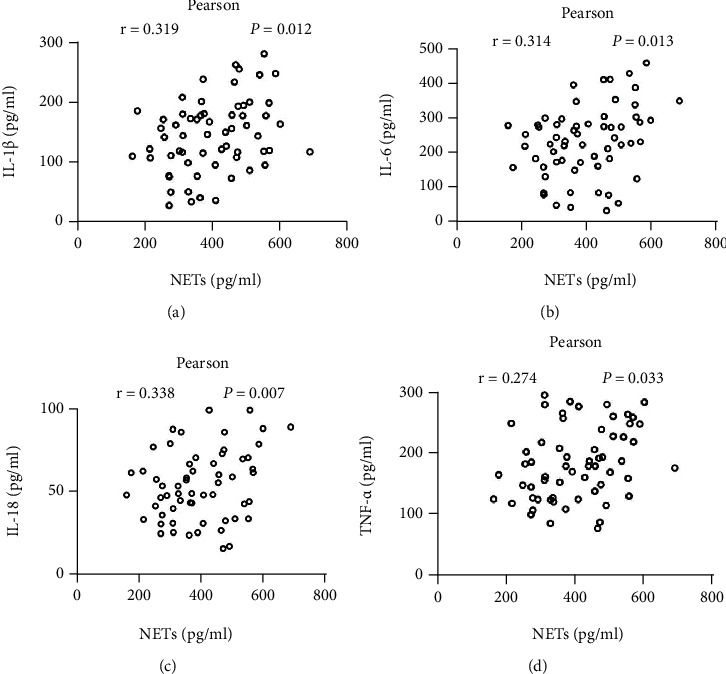
The relationship between inflammatory cytokines and circulating NET levels of lung cancer patients. The circulating NET levels were positively correlated with the levels of the inflammatory cytokines (a) IL-1*β*, (b) IL-6, (c) IL-18, and (d) TNF-*α*.

We further explored the relationship between circulating NET levels and inflammatory cytokines. Increased circulating NET levels were positively correlated with the levels of inflammatory cytokines, including IL-1*β* (Figure 3(a)), IL-6 (Figure 3(b)), IL-18 (Figure 3(c)), and TNF-*α* (Figure 3(d)).

### 3.4. Neutrophils from Lung Cancer Patients Are More Prone to Release NETs

**Figure 4:**
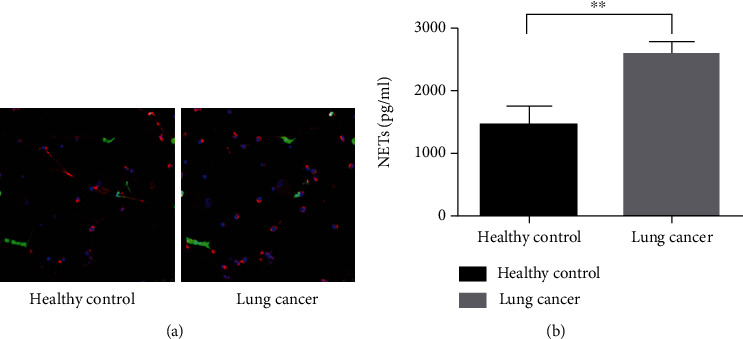
Neutrophils from lung cancer patients are more prone to release NETs. Neutrophils isolated from advanced lung cancer patients and healthy donors were collected. After stimulation with PMA, neutrophils from lung cancer patients showed markedly (a) exaggerated NET release and (b) increased levels of extracellular MPO–DNA complexes. In the confocal images, *in situ* structures of NETs in lung cancer tissues were detected as DAPI (colored by blue), neutrophil elastase (NE, colored by red), and citrullinated histone-3 (citH3, colored by green) colocalization. ^∗∗^*P* < 0.01.

Neutrophils isolated from patients with advanced lung cancer and healthy donors were collected. Neutrophils derived from lung cancer patients demonstrated a significantly enhanced propensity to spontaneously release NETs when compared with healthy controls (Figure 4(a)). Additionally, when stimulated with PMA, a NET activator, neutrophils from patients with lung cancer underwent significantly exaggerated NET release compared with healthy controls, as demonstrated by the increased amount of extracellular MPO–DNA complex (Figure 4(b)). Thus, we found that neutrophils from patients with lung cancer were more prone to NET release.

### 3.5. The Proinflammatory Ability of NETs

**Figure 5:**
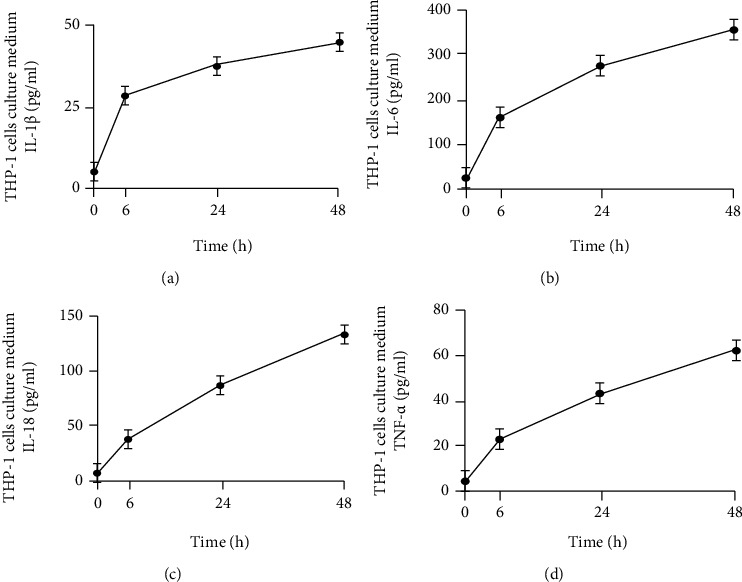
The proinflammatory ability of NETs. Neutrophils were isolated and were incubated with PMA *in vitro*; the supernatants containing the NETs were then collected. The supernatants were used to stimulate THP-1 cells at the indicated time points (0, 6, 24, and 48 h). The levels of (a) IL-1*β*, (b) IL-6, (c) IL-18, and (d) TNF-*α* were measured.

Neutrophils were isolated and incubated with PMA in vitro, and the supernatants containing the NETs were collected. The supernatant was used to stimulate THP-1 cells at the indicated time points (0, 6, 24, and 48 h). Various inflammatory cytokines were also detected. The levels of inflammatory cytokines in the supernatant, including IL-1*β* (Figure 5(a)), IL-6 (Figure 5(b)), IL-18 (Figure 5(c)), and TNF-*α* (Figure 5(d)), increased in a time-dependent manner. The proinflammatory ability of NETs has also been confirmed.

### 3.6. The Contribution of NETs in Promoting A549 Lung Cancer Cell Migration and Invasion

**Figure 6:**
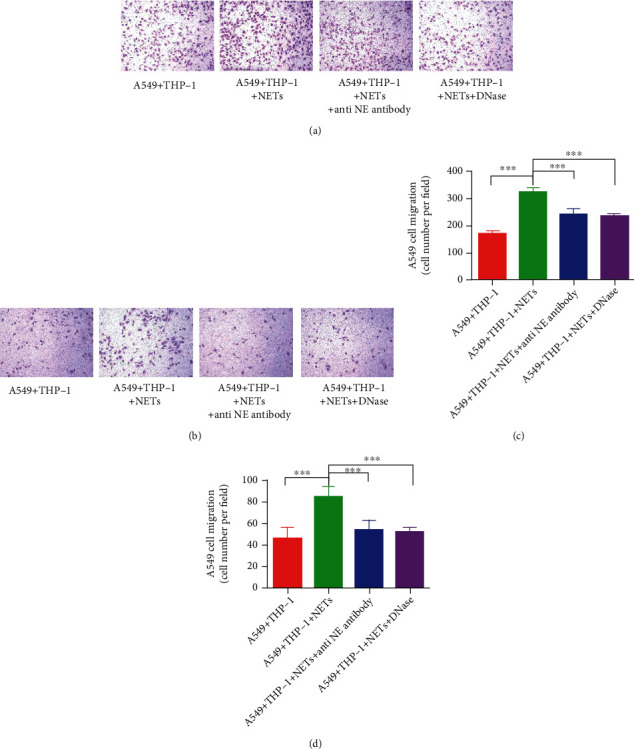
The contribution of NETs in promoting A549 lung cancer cell migration and invasion. To detect the invasion and metastasis abilities of A549 lung cancer cells, Transwell and Matrigel assays were performed. A coculture system of THP-1 and A549 cells was constructed. NETs and NET inhibitors (anti-NE antibody and DNase) promoted the (a, c) migration and (b, d) invasion of A549 lung cancer cells. ^∗∗∗^*P* < 0.001.

The Transwell and Matrigel assays were performed to detect the invasion and metastasis of A549 lung cancer cells. THP-1 and A549 cell coculture systems were also constructed. The supernatant containing NETs promoted migration (Figures 6(a) and 6(c)) and invasion (Figures 6(b) and 6(d)) of A549 lung cancer cells. However, pretreatment with NET inhibitors (anti-NE antibody and DNase) effectively reduced migration (Figures 6(a) and 6(c)) and invasion (Figures 6(b) and 6(d)), which confirmed the ability of NETs to promote migration and invasion of A549 lung cancer cells.

### 3.7. The Ability of NETs to Promote Metastasis and Invasion Is Partly Dependent on Macrophages

**Figure 7:**
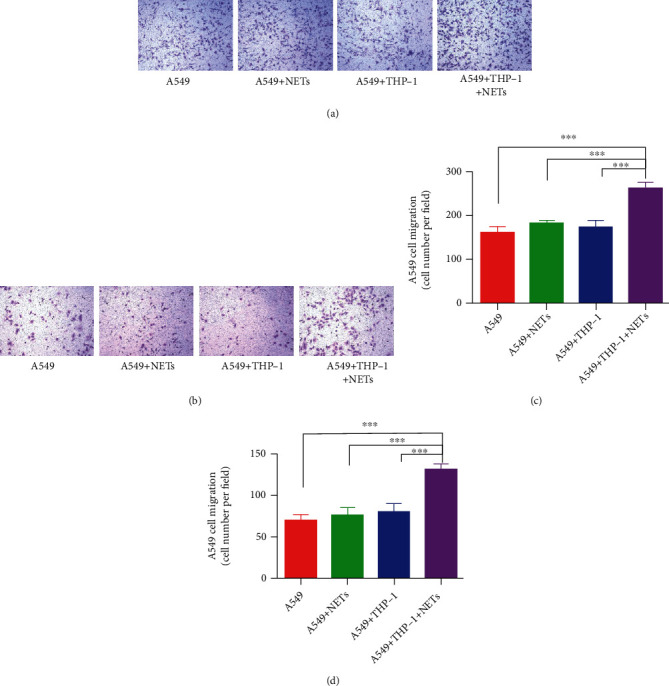
The ability of NETs to promote metastasis and invasion is partly dependent on macrophages. Without THP-1 cells, NETs could not promote A549 cell (a, c) migration and (b, d) invasion. ^∗∗∗^*P* < 0.001.

Without THP-1 cells, NETs did not promote A549 cell migration (Figures 7(a) and 7(c)) or invasion (Figures 7(b) and 7(d)), indicating that the ability of NETs to promote metastasis and invasion is partly dependent on macrophages. Thus, the interaction between macrophages and neutrophils might be involved in the NET-induced migration and invasion of A549 cells. The cytokines released from macrophages may account for this phenomenon. Further in vivo studies are needed to explore the biological functions of NETs in macrophage–neutrophil interactions.

## 4. Discussion

Emerging evidence has confirmed that a variety of immune cells and mediators constitute a complex and dynamic tumor immune microenvironment [20, 21]. As an important part of the immune system, tumor-associated neutrophils are not bystanders [22, 23]. Studies have confirmed that neutrophils are the most predominant leukocyte population in the tumor microenvironment, and the proportion of neutrophils in peripheral blood was closely related to the prognosis of patients with lung cancer [24–26]. NETs are neutrophil-derived webs of DNA decorated with granular proteins, including NE, matrix metalloproteinase 9, MPO, and cathepsin G, with the ability to promote tumor cell proliferation, angiogenesis, and metastasis [27, 28]. Our study also confirmed that NETs participate in the invasion and migration of human lung cancer cell line A549 and further indicated that high circulating NET levels were associated with unfavorable prognosis in patients with lung cancer. To explain the role of NETs in lung cancer, a relevant study proposed that TGF-*β* signals activated by tumor cells induce an overproduction of N1 to N2 neutrophils, which disrupts homeostasis and stimulates tumor invasion [13]. The contribution of NETs to lung cancer cells is also caused by the inhibition of the cells' degradation products on the function of immunosuppressive cells to help tumor cells escape the attack of the immune system [29].

NET formation has been reported to be elevated in lung cancer patients [30]. In our study, the increase in NET formation was positively associated with smoking status, advanced clinical stage, and poor survival. Based on this, circulating NET levels may serve as a biological marker for lung cancer patients with a high risk of progression and metastasis. Moreover, neutrophils from patients with advanced lung cancer are more prone to NET release. We speculate that tumor cells can secrete cytokines to activate neutrophils to form NETs; however, the specific mechanism by which this takes place is unknown. NET release is involved in the chronic inflammation induced by cigarette smoke exposure. Patients with lung cancer are predominantly heavy smokers. We found that heavy smokers had higher levels of circulating NETs. Sustained inflammation caused by tobacco smoke could cause NET formation, which could convert dormant malignant cells to aggressively growing metastases [11]. Thus, NETs may represent a critical mediator between oxidative stress and chronic inflammation induced by cigarette smoke exposure.

Macrophages are the primary contributors to chronic inflammation, respond to danger signals, and rapidly produce large quantities of inflammatory cytokines that enhance the inflammatory response [31]. As an immune cell, macrophages reshape the extracellular matrix, allowing extracellular DNA to enter NETs [32]. The interaction between macrophages and neutrophils may further affect tissue repair [33]. Furthermore, the release of NETs from neutrophils has been shown to license macrophages for inflammatory activation in response to cholesterol crystals during atherosclerosis [34, 35]. However, the mechanism by which the regulatory relationship between macrophages and neutrophils affects tumor progression has rarely been reported. In this study, we confirmed that NETs rely on macrophages to promote the release of inflammatory cytokines, as well as the invasion and metastasis of lung cancer cells. It has been reported that neutrophil-associated chronic low-level inflammation is associated with dysregulation of macrophage-mediated immunosuppression, which together contribute to tumor development [36]. Tumor-associated macrophages also promote the expansion of cancer cells, depending on their functional status [37]. The studies mentioned previously have illustrated the positive roles of NETs and macrophages in promoting the progression of lung cancer. Moreover, in macrophages, the M2 phenotype, as an inhibitor of antitumor immunity, is considered to promote cancer [38]. Therefore, we hypothesized that M2 macrophages may enhance the cancer-promoting mechanism of NETs in patients with lung cancer.

In this study, we also found that increased circulating NETs were positively correlated with the levels of inflammatory cytokines, including IL-1*β*, IL-6, IL-18, and TNF-*α*. A study suggests that IL-1*β* produced by macrophages in the tumor microenvironment promotes tumor growth and metastasis by increasing the expression of novel targets related with angiogenesis and enhancing antiapoptotic signaling in tumor cells [39]. Furthermore, the restoration of IL-6 expression in M2 macrophages enhances migration, chemotaxis, and angiogenesis in lung cancer cells [40]. Lasithiotaki et al. found reduced NLRP3/caspase-1 inflammasome activation and increased IL-1*β* and IL-18 levels in alveolar macrophages in patients with lung cancer [41]. These studies related to inflammatory cytokines support our results and explain how the mechanisms of NETs in promoting lung cancer are related to the increased levels of inflammatory cytokines in macrophages.

As metastasis presents a clinical challenge, the development of therapeutic strategies is important. Since NETs are unable to deplete neutrophils and macrophages, NET formation is a potential therapeutic target. However, some unsolved questions need to be taken into consideration. First, the downstream mechanisms of NET release remain unclear. Second, the effects of NETs in vivo were not assessed. Whether NET inhibitors have a therapeutic effect remains to be confirmed by further studies. Third, the clinical application of NET formation in patients with lung cancer remains unknown. Whether circulating NET levels can be dynamically monitored and serve as a potential prognostic biomarker remains a topic for further study.

## 5. Conclusions

Taken together, our data demonstrated that NETs are involved in the crosstalk between macrophages and neutrophils in the inflammatory microenvironment in lung cancer. A comprehensive and in-depth understanding of the inflammatory microenvironment will be of important theoretical and practical significance for the prevention and treatment of lung cancer. Our findings raise the potential for developing therapeutic strategies to target neutrophil-released NETs. Further larger studies are required to validate these findings and to determine the potential mechanisms underlying NET-associated processes.

## Figures and Tables

**Table 1:** Clinical characteristics of enrolled population.

Characteristics	Lung cancer (*n* = 62)	Health control (*n* = 24)	*P* value
Sex, *n*			
Male	58	18	
Female	14	6	0.562
Age, years (mean ± SD)	58.52 ± 9.78	57.42 ± 11.01	0.655
Smoking history, *n*			
Yes	42	15	
No	20	9	0.645
Tumor size, *n*			
T1	10		
T2	24		
T3	11		
T4	17		
Lymph node metastasis, *n*			
N0	13		
N1	13		
N2	23		
N3	13		
TNM stage, *n*			
I	5		
II	11		
III	19		
IV	27		
Pathology stage, *n*			
Adenocarcinoma	32		
Squamous cell carcinoma	30		
Main driver gene mutation			
EGFR	8		
ALK	1		
Peripheral leukocyte	7.91 ± 2.86	7.38 ± 1.15	0.153
Peripheral neutrophils	5.60 ± 2.75	5.27 ± 1.79	0.437

SD: standard deviation; EGFR: epidermal growth factor receptor; ALK: anaplastic lymphoma kinase.

## Data Availability

The raw data, supporting the conclusions of this manuscript, will be made available by contacting the corresponding author.
